# Molecular Chaperonin HSP60: Current Understanding and Future Prospects

**DOI:** 10.3390/ijms25105483

**Published:** 2024-05-17

**Authors:** Manish Kumar Singh, Yoonhwa Shin, Sunhee Han, Joohun Ha, Pramod K. Tiwari, Sung Soo Kim, Insug Kang

**Affiliations:** 1 Department of Biochemistry and Molecular Biology, School of Medicine, Kyung Hee University, Seoul 02447, Republic of Korea; manishbiochem@gmail.com (M.K.S.); jac03032@khu.ac.kr (Y.S.); sunheehan@khu.ac.kr (S.H.); hajh@khu.ac.kr (J.H.); 2 Biomedical Science Institute, Kyung Hee University, Seoul 02447, Republic of Korea; 3 Centre for Genomics, SOS Zoology, Jiwaji University, Gwalior 474011, India; pk_tiwari@hotmail.com; 4 Department of Biomedical Science, Graduate School, Kyung Hee University, Seoul 02447, Republic of Korea

**Keywords:** chaperonin, molecular chaperone, cancer, HSP60, inflammation, mitochondria, protein folding, apoptosis

## Abstract

Molecular chaperones are highly conserved across evolution and play a crucial role in preserving protein homeostasis. The 60 kDa heat shock protein (HSP60), also referred to as chaperonin 60 (Cpn60), resides within mitochondria and is involved in maintaining the organelle’s proteome integrity and homeostasis. The HSP60 family, encompassing Cpn60, plays diverse roles in cellular processes, including protein folding, cell signaling, and managing high-temperature stress. In prokaryotes, HSP60 is well understood as a GroEL/GroES complex, which forms a double-ring cavity and aids in protein folding. In eukaryotes, HSP60 is implicated in numerous biological functions, like facilitating the folding of native proteins and influencing disease and development processes. Notably, research highlights its critical involvement in sustaining oxidative stress and preserving mitochondrial integrity. HSP60 perturbation results in the loss of the mitochondria integrity and activates apoptosis. Currently, numerous clinical investigations are in progress to explore targeting HSP60 both in vivo and in vitro across various disease models. These studies aim to enhance our comprehension of disease mechanisms and potentially harness HSP60 as a therapeutic target for various conditions, including cancer, inflammatory disorders, and neurodegenerative diseases. This review delves into the diverse functions of HSP60 in regulating proteo-homeostasis, oxidative stress, ROS, apoptosis, and its implications in diseases like cancer and neurodegeneration.

## 1. Introduction

In biological systems, stress and the stress response are evolutionarily conserved processes. The stress response and various stress proteins are well-characterized from prokaryotes to eukaryotes [1]. Heat shock proteins (HSPs), also referred to as molecular chaperones, facilitate the folding of misfolded proteins and aid in protein folding, trafficking, assembly, and degradation, resulting in the maintenance of cellular protein homeostasis [2,3]. HSP60, a 60 kDa molecular chaperonin primarily described as a GroEL/GroES system in *Escherichia coli* In humans, HSP60 chaperonin(s), also known as Cpn60/HSPD1, has an important and passive function in the folding of naïve polypeptides and unfolded proteins in the cytoplasm and mitochondria [4]. HSP60 consists of two major subgroups of chaperonins: Group I chaperonins, also known as HSPD1, are found in eubacteria and organelles of endosymbiotic origin, such as mitochondria and chloroplasts [5], while prokaryotes cooperate with cofactors of the GroES or HSP10 family of proteins, GroEL. Group II chaperonins, expressed in the eukaryotic cytosol, are HSP10-independent. However, chaperonin function requires the binding and release of ATP within the ring structure. The non-native substrate proteins are captured through hydrophobic interactions at the inner cavity and are then entrapped in the central ring cavity, where they are folded and protected from further aggregation [5,6]. HSP60 showed significant structural and functional similarities across species from *E. coli* to humans. However, the different variants of the HSP60 gene and their isoforms suggested that the HSP60 gene evolved from prokaryotes and sub-cellular organelles such as mitochondria [7]. HSP60 is a conserved protein and structurally forms a large double-ring barrel-like structure, shaping a central cavity with a closed lid formed with a co-chaperone GroES or HSP10, involved in the folding of native peptides and misfolded proteins. After correct folding, the folded protein is released into the cytoplasm [8]. As a mitochondrial chaperone (mtHSP60), its function is reported in the regulation of oxidative and free radical stress and protects the cells from apoptosis. Moreover, HSP60 functions are ambiguous in human diseases and stress conditions as compared to other heat shock proteins such as HSP70, HSP90, and small HSPs. This review highlights the important features and functions of HSP60 in cellular homeostasis and human diseases. The major focus of this review is discussing the structure of HSP60, its co-chaperones, its sub-cellular localization, and its diverse function in immunity, cancer, and various diseases for future therapeutics.

## 2. HSP60 Structure and Functions

### 2.1. HSP60 Molecular Characteristics and Regulation

**Figure 1:**
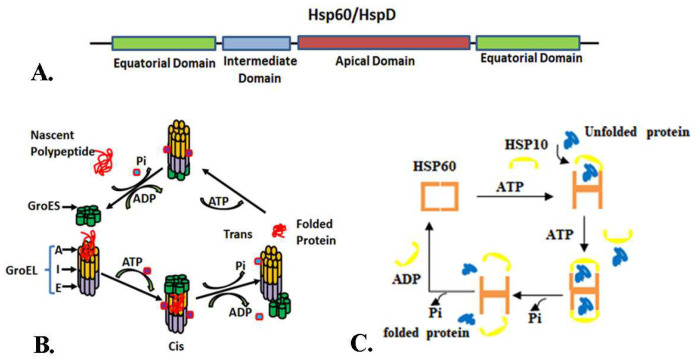
The diagram illustrates the domain arrangement of the HSP60 protein, showcasing its bacterial form, GroEL, and its eukaryotic counterpart, HSP60. (**A**) The HSP60/GroEL structure features three principal domains: equatorial, intermediate, and apical. This protein folding process involves ATP-dependent folding of newly synthesized or misfolded proteins, facilitated by GroEL/GroES in bacteria (**B**) and HSP60/HSP10 in humans (**C**). ATP activation plays a crucial role in transitioning proteins between cis and trans conformations. Upon ATP hydrolysis, GroES/HSP10 dissociates from GroEL/HSP60, allowing the release of properly folded proteins into the cytoplasm.

The HSP60 is a double-ring assembly ubiquitously expressed across prokaryotes to eukaryotes. It is a nuclear-encoded protein synthesized in the cytoplasm and transported into the sub-cellular organelles; thus, it is also known as a molecular chaperonin. HSP60 is localized in both mitochondrial and cytoplasm, 80–85% being mitochondrial and the remaining 15–20% in the cytoplasm [9]. The HSP60 family comprises *E. coli* homolog GroEL (large) and GroES (small), Cpn60 (60 kDa chaperonin), CCT (chaperonin-containing TCP-1), and TRIC (TCP-1 ring complex) in mammals, with mitochondrial mtHSP60 being another prominent member. HSP60 is categorized into two groups: Group I chaperonins and Group II chaperonins [8]. Unlike bacteria, archaea lack the Group I chaperonin system. Instead, they possess the HSP70 (Dnak) chaperonin. The absence of the Group I chaperonin in archaea is intriguing since, in bacteria, the two systems coexist and seem to have evolved together. In prokaryotes, the naive polypeptide is transported to the GroEL/GroES complex for final folding [10]. An archaeal chaperonin complex resembles a eukaryotic cytosolic protein called TCP1 (tailless complex polypeptide-I), CCT, or TRiC (Table 1) [11,12]. TRiC/CCT consists of two oligomeric rings, each composed of eight homologous subunits, forming a central cavity, which serves as a place for protein client folding [13,14,15,16]. Structurally, HSP60 comprises two heptameric rings consisting of 14 subunits, with each subunit being approximately 60 kDa. In eukaryotes, HSP60 forms a hetero-oligomeric complex; however, in prokaryotes, it forms a homo-oligomer structure [17]. Under physiological conditions, the cylindrical structure harbors a large cavity formed by two rings that assist in chaperoning function. In prokaryotes, such as *E. coli*, the GroEL monomer consists of three distinct domains: the apical domain forming the barrel, the intermediate domain connecting the other two domains, and the equatorial domain at the base of the ring (Figure 1). GroES, on the other hand, is a single ring serving as a lid to occlude one end of the GroEL barrel, forming an asymmetrical complex [18]. In an asymmetric cycle, the client protein undergoes folding within the cavity during ATP hydrolysis. Concurrently, the open trans ring can bind a non-native substrate, and upon ATP hydrolysis in the cis ring, ATP recruitment to the trans ring triggers the dissociation of ADP and GroES. This event enables the release of client protein. Symmetrical cis complexes, when compared to asymmetric trans complexes, are noted to be unstable [18,19]. The GroEL/GroES complex in prokaryotes aids in the correct folding and assembly of post-translated proteins, particularly major oligomeric proteins requiring assistance for proper folding. This process involves the association of these proteins with the chaperonin complex, although they are not themselves part of the complex. Under pathological conditions, there is a marked increase in HSP60, which can exhibit either pro-survival or lethal functions, contingent upon whether it exists in a monomeric form or oligomeric form. An intriguing hypothesis posits that the oligomerization state of HSP60 may be associated with its pathological or functional role outside the mitochondria [17,20]. 

Archaeal chaperonins, akin to those found in eukaryotes, were traditionally believed to comprise two subunits. However, recent findings have unveiled the presence of a third subunit in CCT, suggesting a more complex assembly. Notably, this additional subunit exhibits thermostable ATPase activity and is observed to accumulate following exposure to high temperatures. Studies conducted on *T. acidophilum*, an archaeon, have elucidated that these chaperonins consist of two domains hosting alternate subunits, denoted as α and β, each approximately 58 kDa in size. Upon encapsulation of native proteins within the cage, the binding of ATP initiates numerous conformational changes. These changes facilitate the conversion of hydrophobic conformations to hydrophilic ones. Consequently, the enclosed polypeptide undergoes folding or refolding and is subsequently released into the cytoplasm after refolding. The lid domain tightly interacts with the apical domain, akin to the cis-ring of the GroEL–GroES complex. Interestingly, in extreme halophiles like *H. valcanii*, belonging to the phylum Euryarchaeota, homologous genes named cct1, cct2, and cct3 have been identified, resembling the eukaryotic CCT gene [21]. Notably, while the eukaryotic CCT is non-heat inducible, its archaeal counterpart demonstrates heat inducibility. The arrangement of highly charged residues in the cis configuration potentially plays a pivotal role in substrate interaction. Conversely, in the trans configuration, conserved charged residues located within domains A and I interposed between monomers facilitate hydrophobic interactions crucial for substrate binding. Apart from morphological disparities, Group I and Group II chaperonins diverge in functional aspects, highlighting the intricate diversity within chaperonin systems across different domains of life.

In eukaryotes, HSP60 initially exists as a heptameric single-ring structure in the absence of ATP. However, its interaction with HSP10 required the presence of ATP, leading to the formation of a double-ring structure (Figure 1). Furthermore, it is noteworthy that HSP60 does not bind with HSP10 in the presence of ADP, while GroEL is capable of binding with GroES even in the presence of ADP [22,23]. Wang et al. elucidated the structural properties of the mitochondrial HSP60 (mHSP60) and the specificity of its subunits. The dynamics of mHSP60 regarding its associations are primarily driven by unique sequences at the inter-subunit interface. Specifically, residues T35, S57, D59, V112, A516, and E522 exhibit conservation within mitochondrial chaperonins across higher eukaryotes, including humans, and are also present in lower organisms like yeast, resembling those found in GroEL. Additionally, yeast feature sequences for one of the two conserved inter-ring salt bridges observed in GroEL, namely R452-E462, while higher eukaryotes lack sequences for both salt bridges. The influence of mHSP10 on the quaternary structure of mHSP60 underscores the significance of structure dynamics within the heptameric mHSP60 complex, highlighting mechanistic disparities between mHSP60–mHSP10 and GroEL–GroES systems [24].

CCT exhibits the capability to bind and facilitate ATP-dependent folding not only of actin and tubulin. A recent structural analysis has revealed that actin monomers lack the ability to spontaneously fold and are transiently trapped in an unstable intermediate state. Binding to TRiC/CCT is critical for achieving the native folding state of actin, as confirmed by the deletion of CCT subunits leading to the disorganization of actin filaments [25,26]. Despite its crucial roles in regulating actin folding and cytoskeletal architecture, its involvement in the regulation of epithelial or endothelial barriers remains poorly understood. For instance, in *C. elegans*, the loss of the CCT5 subunit caused a significant disorganization of the apical actin cytoskeleton, accompanied by abnormal morphology of the apical plasma membrane and decreased microvilli length [27]. However, despite these epithelial organization defects, no significant increase in intestinal permeability to large molecules (such as Texas red dextran) was observed [27]. Nevertheless, there has been no thorough examination of gut barrier structure and permeability in CCT5-null animals. Additionally, the overexpression of various CCT subunits is crucial for numerous solid tumors [28,29]. This heightened expression significantly contributes to the promotion of tumor cell invasion and metastasis, playing a causal role in these processes. Furthermore, the functions of TRiC/CCT at the apical junction complex are not well characterized. 

In cancer cells, overexpression of HSP60 is regulated by the proto-oncogenes c-MYC and HSF1. HSP60 transcriptionally activates ClpP via *c-Myc* and physically interacts with ClpP in mitochondria via its apical domain. HSP60 promotes β-catenin signaling by maintaining ATP production, resulting in *c-Myc* upregulation. Additionally, the HSP60–ClpP interaction is responsive to the mitochondrial unfolding protein reaction (UPR^mt^). UPR^mt^ activation involves multiple transcription factors, notably activating transcription factor 5 (ATF5), activating transcription factor 4 (ATF4), and C/EBP homologous protein (CHOP) localized in mitochondria under normal conditions, translocate to the nucleus alongside CHOP and ATF4 under malignant stress stimuli such as ROS and damaged mitochondrial DNA, synergistically promoting HSP60 overexpression [30]. HSF1 and single-stranded DNA-binding protein 1 (SSBP1) form a complex that binds to the promoter region encoding the mitochondrial chaperone, subsequently recruiting Brahma-related gene 1 (BRG1), a chromatin regulating factor, thereby enhancing the overexpression of HSP60 [31]. Notably, the binding of STATE3 to the promoter also induces the expression of HSP60. However, the inhibition of HSP60 expression can result from the perturbation of STAT3 binding [32].

HSP60 expression can be regulated by miRNA, members of non-coding RNA, which negatively regulates gene expression by binding to the target mRNA. Studies have shown that miR-1 and miR-206 target HSP60 mRNA, inhibiting HSP60 expression and accelerating cardiomyocyte apoptosis [33]. Furthermore, miR-17a has been shown to regulate the expression of HSP60 in gastric lymphoma. Elevated expression of miR-17 has been observed in gastric lymphoma, contributing to tumor development, progression, and metastasization by modulating the HSP60/TNFR2 pathway [34]. The expression of HSP60 is regulated at various levels, encompassing transcription, translation, and post-translation modifications (PTMs). The HSP60 amino acid sequence undergoes various PTMs, including GlcNacylation, nitration, acetylation, S-nitrosylation, citrullination, methylation, oxidation, biotinylation, and ubiquitination [35]. These modifications are context-dependent and have implications for various human diseases [36]. Specific phosphorylation sites, including Ser70, Tyr227, and Tyr243 of HSP60, play crucial roles in cancer progression and growth. For instance, acetylation of HSP60 not only promotes its degradation but also facilitates the degradation of p53, suppressing cancer growth [37,38]. Also, HSP60 nitration can impair mitochondrial function and contribute to its translocation to exosomes for secretion outside the cells. The roles of PTMs, such as ubiquitination, methylation, and O-GlcNAcylation, in tumors remain to be fully explored [35]. The above-mentioned findings highlight the pivotal role of miRNA in regulating HSP60 expression in diverse tissues and tumor types, offering promising avenues for innovative cancer therapeutics. Furthermore, the use of exosome-mimicking vesicles for drug delivery, which regulate HSP60 and enhance cellular uptake, underscores the need for proactive research to standardize these particles and their contents, along with their isolation from cancer patients. 

### 2.2. HSP60 Transcript Variants and Subcellular Localization

HSP60 genes, including isoforms named *HSP60A, HSP60B, HSP60C*, and *HSP60D*, were identified subsequent to the completion of the Berkeley *Drosophila* genome project [39,40]. Although HSP60 was initially thought to be non-heat-inducible, subsequent studies revealed that *HSP60A* expression can indeed be triggered under high-temperature conditions in *Drosophila* embryos, indicating post-transcriptional regulation. The expression pattern of *HSP60A* is critical for maintaining protein composition and mitochondrial biogenesis during embryogenesis, tailored to the specific energy demands of different cell types [41]. Interestingly, the regulation of HSP60 during embryonic stages in *Drosophila* is dynamic and influenced by stimuli. Unlike mRNA levels, which are evenly distributed throughout early embryonic stages, the protein level of HSP60 shows significant variation, with a notable upregulation in whole embryos and a decrease at temperatures exceeding 40 °C [42]. In *Tetrahymena* sp., HSP60 protein levels increase two- to three-fold upon heat shock, while in sea anemones, *A. viridis*, HSP60 levels are significantly induced at high temperatures compared to normal temperatures [43]. The chaperonin activity of HSP60 is crucial for cellular protection against thermal injury. This hypothesis finds support in the cytoprotective role of HSP60 under high-temperature stress or exposure to chemical pesticides, indicating its potential role in cellular protection as well as stress-induced damage [44,45,46]. Another variant, *HSP60B*, has been implicated in male fertility in *Drosophila*, particularly in sperm individualization. Studies in *Drosophila* germ cells have demonstrated the necessity of *HSP60B* for male fertility [47,48]. Similar results were also detected in spermatogonia and spermatocytes in rodents [49] and humans. Additionally, HSP60 is localized on the acrosome of mouse epididymal sperm within the epididymal epithelium and dense bodies, indicating its involvement in spermatogenesis and fertilization [49,50]. Further, *HSP60C* is reported in tracheal development and male fertility [51]. Another variant, *HSP60D*, has been revealed to have a dual role in pro- and anti-apoptotic pathways in *Drosophila* [52]. In humans, homologs of HSP60 have been reported to play a role as anti- or pro-apoptotic, similar to HSP60D [53,54]. Chandra et al. reported that cytosolic accumulation of HSP60 activates apoptosis through its interaction with procaspase-3, leading to the subsequent maturation and activation of caspase-3 [53]. 

In pathological conditions, HSP60 accumulates in extra-mitochondrial locations, where it can exert either pro-survival or pro-death functions [53,55]. Surface-bound HSP60 might be involved in membrane transport and signaling. An upsurge of HSP60 on plasma membranes serves as a danger signal for the immune system, triggering the activation and maturation of dendritic cells and eliciting an antitumor T-cell response [56]. Gamma-delta T lymphocytes recognizing HSP60 facilitate the lysis of oral tumor cells [57]. Conversely, surface-associated HSP60 contributes to the metastasis of pancreatic carcinoma [58]. Thus, HSP60 can exhibit contrasting effects on tumor cell survival; in certain malignancies, it plays a cytoprotective role, while in others, it promotes apoptosis. Additionally, a study by Sarangi et al. revealed that HSP60 acts as a barrier against mitochondrial oxidative stress-mediated apoptosis in cancer cells [54]. In humans, HSP60 shares the highest level of identity with mouse chaperonin, indicating the possibility of the existence of transcript variants of HSP60 in various tissue types of different species. Furthermore, missense mutations in genetic variants of hHSP60 are associated with multiple disorders in humans. Specifically, SPG13, pV98I, and pQ461E missense mutations are reported in spastic paraplegia [59,60,61]. Another missense mutation, HDL4, caused by pD29G, is associated with hypomethylating leukodystrophy [62,63,64]. Notably, cardiomyocytes release exosomes containing HSP60 when they are exposed to mild stress conditions. HSP60 has also been detected in various tumors and is highly expressed and secreted, suggesting its potential as a biomarker in various cancers when present in biological fluids such as plasma serum [65]. Thus, HSP60 serves as a candidate for monitoring disease progression and response to treatments, with great advantages in non-invasive biopsies [65]. Understanding the diversity and functions of HSP60 transcript variants provides valuable insights into the molecular mechanisms underlying cellular homeostasis and organismal health, potentially offering parallel observations in humans.

Although HSP60 is primarily localized in mitochondria, in silico studies have suggested its presence outside mitochondria, including in the endoplasmic reticulum (ER) and on the cell surface [44,66]. While HSP60 has been implicated in diverse cellular functions within mitochondria and the cytoplasm of multicellular organisms, its specific sub-cellular localization, such as the ER, nucleus, and cell surface, remains unclear. Research indicates HSP60 localization in the polytene nuclei of various tissues in invertebrates, hinting at its nuclear involvement [44]. Expression studies in larval polytene cell types suggest HSP60 primarily resides in the cytoplasm. However, in somatic tissues like fat bodies (FBs) and Malpighian tubules (MTs), HSP60 appears to localize to the nucleus, implying potential nuclear functions. Some studies have also noted HSP60’s presence in the nucleus [67,68]. Despite lingering ambiguity regarding HSP60’s nuclear localization, further investigation is necessary to confirm its translocation from mitochondria to the nucleus and other cytoplasmic locations such as the ER and cell surface. Additional research may shed light on its unknown functions in the nucleus and other sub-cellular organelles.

## 3. HSP60’s Role in Development and Differentiation 

HSP60 has shown a dynamic pattern across various somatic tissues such as the fat body (FB), Malpighian tubules (MTs), salivary gland (SG), and germinal cell oocytes in several insects, including *Drosophila*, suggesting that the regulation of HSP60 protein expression varies depending on the cell type and response to thermal protection mechanisms [46]. In situ hybridization and quantitative mRNA analyses in various tissues and different stages of oocyte development align with previous studies, indicating that the thermal response of HSP60 is largely independent across larval and adult developmental stages of *Drosophila* [69]. HSP60-induced expression is also observed during the developmental stages of locusts, leaf miners, and *Drosophila* [70,71,72]. In the blowfly, *Lucilia cuprina*, induced expression of HSP60 is observed in various stages of developing oocytes (1st–14th stages). The distribution of HSP60 expression near nurse cells and the follicular layer, later moving to the oocyte region, suggests both developmental and protective roles for HSP60 in oocytes. Intriguingly, HSP60A has shown dynamic and stage-specific variations in expression patterns across early embryogenesis, which are post-transcriptionally regulated, having a role in mitochondrial biogenesis to meet energy demands in different cell types during embryogenesis [42,73]. Mutational studies in *Drosophila* have shown HSP60B to be essential for male germ line development, particularly in the spermatid individualization process [48]. Besides embryonic functions, HSP60 plays a crucial role in the differentiation of the epithelial cells within the mammalian gut. The deletion of the HSP60D1 gene either in the intestinal epithelial or intestinal stem cells results in the loss of cell stemness, reduced cell proliferation, and abnormal development of Paneth cells [74,75]. These anomalies are associated with the known localization of mammalian HSP60 within mitochondria and its participation in mitochondrial functions [76]. Knockdown of HSPD1 in intestinal epithelial cells results in mild mucosal inflammation, potentially indicating a disruption in gut barrier function [74]; however, neither barrier permeability nor the integrity of intestinal epithelial junctions are explored in animal models. 

Furthermore, depletion of HSP60 in mouse embryonic cells has been shown to inhibit embryonic stem cell (ESC) proliferation and increase apoptosis during embryoid body (EB) differentiation. This highlights its crucial role in maintaining ESC stemness and facilitating differentiation [77]. Similarly, in human pluripotent stem cells (hPSCs), depletion of HSP60 results in decreased expression of pluripotent genes such as OCT4, NANOG, and SOX2, and naïve-state-specific genes including OTX2, KLF4, and DNMT3B in naïve hPSCs [78]. Notably, HSP60 is found on the cell surface in these cells, suggesting its essential role in maintaining naïve pluripotency and promoting hPSC differentiation. The presence of HSP60 on the cell surface may involve additional post-translational modifications or conformational changes that require further exploration [35]. In multiple myeloma (MM) CD138+ plasma cells (PCs), HSP60 expression is notably elevated. However, knockdown of HSP60 leads to metabolic rearrangements and inhibition of myeloma cell proliferation [79]. These findings suggest that HSP60 could serve as a potential target for reducing cell proliferation in myeloma, with further research potentially informing clinical applications.

## 4. HSP60’s Role in Various Human Diseases

**Figure 2:**
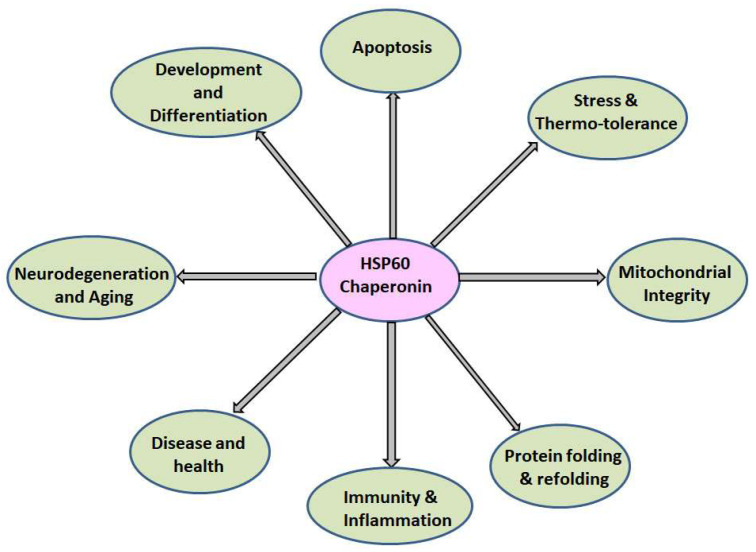
The diagram illustrates HSP60’s engagement in diverse biological functions, depicting various cellular pathways such as mitochondrial integrity, apoptosis, protein folding, inflammatory responses, and disease management. HSP60 plays a pivotal role in maintaining protein homeostasis and thereby ensuring the functionality of crucial processes like development and stress adaptation and maintaining cellular integrity. Moreover, HSP60 influences inflammatory pathways, immune response, and apoptosis in various human diseases, including cancer, aging, cardiomyopathy, arthritis, neurodegeneration, and so on.

HSP60, aside from its constitutive function in nascent polypeptide folding, plays a crucial role in various cellular processes, including stress regulation, inflammation activation, apoptosis, pathogenic infections, neurodegeneration, and cancers. While it is constitutively expressed under normal conditions, its expression increases notably in pre-neoplastic lesions and various diseases [80,81]. Cytoplasmic HSP60 is involved in cell signaling in diverse cell types, such as cardiac myocytes and hepatocytes [82]. Cytosolic HSP60 promotes the TNF-α-mediated activation and phosphorylation of NF-kB kinases (IKKs), thereby protecting cells from mitochondrial-derived oxidative stress by modulating NF-kB targeted gene expression [83,84,85]. Mitochondrial HSP60 primarily assists in the folding, refolding, and degradation of mitochondrial proteins [54,86,87]. HSP60 also protects cardiac myocytes during hypoxia by forming complexes with Bax, a pro-apoptotic protein, thereby preventing its translocation from the inner mitochondrial membrane. Moreover, HSP60 protects epithelial cells from stress-induced cell death by activating extracellular signal-regulated kinases (ERKs) and inhibiting caspase-3 [88]. Elevated levels have been linked to several diseases, including type 2 diabetes, hepatitis B, cardiovascular disease, atherosclerosis, periodontitis, juvenile idiopathic arthritis, and various cancers (Figure 2) [89,90,91,92].

### 4.1. Role of HSP60 in Metabolic Diseases

Stressors of various kinds, including chemical, biological, and environmental factors, upregulate the expression of stress proteins. In diabetic conditions, inhibition of the MEK/ERK signaling pathway inhibits insulin-induced transcription of the HSP60 gene, resulting in the accumulation of HSP60 and emphasizing the insulin-dependent regulation of HSP60. Thus, HSP60 is negatively regulated by insulin [93]. In vitro experiments on HeLa cells exposed to high glucose and H_2_O_2_ demonstrate elevated levels of HSP60 and oxidative stress, suggesting its association with type 2 diabetes (T2DM) and neuroinflammation. Additionally, in diabetic patient saliva and serum samples, HSP60 levels are upregulated, reinforcing its role in T2DM. In type 2 diabetes, insulin resistance and increased glucose levels cause mitochondrial dysfunction and decreased expression of HSP60 in the brain, which promotes brain hyperglycemia, resulting in high ROS levels and contributing to mitochondrial dysfunction [94]. This highlights the importance of HSP60 expression in type 2 diabetes for maintaining mitochondrial proteostasis. Notably, the interaction between HSP60 and toll-like receptors (TLRs) supports the hypothesis that HSP60 could be involved in disease progression. However, the dysregulation of HSP60 in mouse hypothalamic cell lines and brain samples from T2DM patients further supports its role in the pathophysiology of T2DM [95,96]. Recently, it has been reported that in burn sepsis situations, mitochondrial energy metabolism is restored in hepatocytes through upregulation and activation of SIRT4, an NAD+-dependent deacetylase, which subsequently reduces acetylation of HSP60 and facilitates the assembly of the HSP60–HSP10 complex. This reduced acetylation of HSP60–HSP10 restores the mitochondrial energy metabolism at complex II and complex III via glutamine-mediated SIRT4 activity (Table 2). Therefore, HSP60 may serve as a target of SIRT4 in hepatocytes, improving ATP production and protecting from burn-sepsis-dependent organ injury [97]. The reduction in HSP60 modulates the expression of AKT and p53 through SIRT1 activation induced by resveratrol in HEK293 T cells [98]. Further exploration of HSP60 may provide insights into insulin-resistant and mitochondrial functions, directing attention towards its role in metabolic diseases and aiding in the development of new therapeutic strategies.

### 4.2. Role of HSP60 in Infectious Diseases

Circulating HSP60 levels are associated with both pro-inflammatory and anti-inflammatory activities. In autoimmune diseases, the expression of HSP60 on the cell surface is upregulated following stimulation by environmental factors. Bacterial HSP60 (GroEL) can induce an immune response in the host tissues and produce auto-HSP60 antibodies. These autoantibodies can cross-react with and bind to HSP60 on the surface of epithelial cells, forming immune complexes. This process can contribute to the apoptosis of endothelial cells in various autoimmune diseases, such as immune glomerulonephritis and vasculitis, including Behcet’s disease, Takayasu’s arteritis, and various other autoimmune diseases [99,100]. HSP60 displays similarity to various other proteins known to elicit immune responses, such as AChRα1, Chlamydia pneumoniae GroEL, or Chlamydia trachomatis GroEL, in Myasthenia gravis (MG), a neuromuscular disorder. Consequently, autoantibodies targeting self-HSP60 could serve as potential targets for both the treatment and early detection of MG [129]. *H. pylori*, a gram-negative bacterium, is associated with several gastrointestinal diseases such as gastritis, gastric ulcer, and gastric cancer (GC). Studies on extracellular vesicles (EVs) from gastric epithelial cells have revealed various cytotoxic factors, such as cytotoxin-associated gene A (CagA) and cytokines, which suppress T-cell activity. Analysis of EVs from the blood samples of H. pylori-infected patients has shown differentially expressed various proteins, including upregulation of HSP60 in gastric cancer and gastric ulcer tissues. The expression of HSP60 progressively increases from gastric dysplasia and gastric cancer, as confirmed by histopathological analysis of patients’ samples across various grades [101]. Deletion of HSP60 in EVs results in reduced expression of Bcl2, an anti-apoptotic protein, while enhanced expression of the apoptotic protein Bax promotes cell apoptosis. This suggests that HSP60 may regulate apoptosis during *H. pylori* infection [102]. Further investigation is warranted to gain more insight into the potential role of HSP60 in *H. pylori* infection and gastric diseases. In human immunodeficiency virus (HIV) patients, HSP60 levels are elevated, which results in an increased risk of cardiovascular diseases due to elevated levels of CD4 and circulating CD14 levels induced by viral load. Additionally, in vitro studies have shown that elevated HSP60 levels induce apoptosis in rat osteoblast cells and increase bone absorption [130]. Thus, an elevated level of HSP60 has a high risk of osteoporosis in HIV-infected patients [131]. In prokaryotes, HSP60 expression is induced as part of an immune response in many diseases. In humans, conjunctivitis induces an anti-inflammatory response against bacterial HSP60 from *C. trachomatis* [132]. HSP60 regulates the replication of foot-and-mouth disease virus (FMDV) viral RNA (vRNA) replication and mRNA translation via binding to 2C and 3A proteins, thereby maintaining their stability through the inhibition of autophagy–lysosomal and apoptosis pathways. Depletion of HSP60 potentially diminishes FMDV pathogenicity in infected mice [103]. This study unveils a novel role for HSP60 in FMDV replication and offers insights that could aid in the development of therapeutics targeting viral infections. 

### 4.3. Role of HSP60 in Cardiovascular Diseases and Atherosclerosis

Particularly, within the spectrum of vascular diseases, both TLRs and HSPs play pivotal roles in regulating cellular signaling. The HSP60 protein functions have been studied in cardiovascular diseases via toll-like receptors (TLRs). Among the ten TLRs identified in humans, TLR2 and TLR4 have been specifically linked to the pathogenesis of cardiovascular ailments [104,105]. In vitro studies conducted in HEK293 cells have demonstrated that HSP60-induced proliferation of venous smooth muscle cells (VSMCs) is mediated through the activation of TLR4 and TLR2 pathways [133]. Knockout transgenic mice lacking HSP60 showed dilated cardiomyopathy and heart failure due to disrupted mitochondrial protein homeostasis and function [106]. In patients with cardiovascular disease and acute myocardial infarction, serum HSP60 levels have been elevated compared to control, indicating its potential utility as a diagnostic marker for disease progression [106]. HSP60 present within exosomes represents a promising target for attenuating apoptosis in cardiomyocytes. For instance, in mice, following infection with Coxsackievirus B3 (CVB3), the expression of cathelicidin-related antimicrobial peptides (such as CRAMP and LL-37) increases. This leads to the release of exosomes containing a variety of viral and host components, thereby facilitating viral replication induced by CVB3 infection [134]. CRAMP directly targets exosomal HSP60, thereby impeding exosome-mediated CVB3 transmission and inhibiting HSP60-induced apoptosis and CVB3 replication. This investigation underscores the role of exosomes harboring HSP60 in the pathogenesis of acute viral myocarditis, highlighting the potential efficacy of targeting these exosomes to restrict viral infection [135]. Another study has been demonstrated through an analysis of *Chlamydia pneumonia* infection and its relation to atherosclerosis. This infection serves as a stress factor to the endothelium, promoting the release of human HSP60 (hHSP60) into the cytosol and its expression on the endothelial surface. This process aids in initiating autoimmune reactions initiated by cHSP60 [136]. Additionally, cHSP60 stimulates the specific proliferation of T lymphocytes and B lymphocytes, resulting in increased secretion of IL-10 and IL-6 (Table 2) [137]. Consequently, cHSP60 and hHSP60 are found in plaques in carotid atherosclerotic plaque samples, playing a role in promoting TNF-α secretion [107]. In cardiovascular disease (CVD), elevated levels of HSP60 lead to the production of anti-HSP60 autoantibodies, which induce cellular cytotoxicity of stressed endothelial cells. In vivo pretreatment of ApoE^−/−^ mice with the F(ab)2 fragment of the IL-13 monoclonal antibody, targeting the 288–366 epitope site on HSP60, decreases the growth of the atherosclerotic lesions [108,138]. These research findings indicate that HSP60 is a promising target for developing an early marker for cardiovascular diseases. 

### 4.4. Role of HSP60 in Inflammatory Diseases

HSP60 plays a pivotal role in both innate and adaptive immunity, regulating the secretion of pro-inflammatory and anti-inflammatory cytokines in various human diseases. HSP60 activates endothelial cells, smooth muscle cells, and monocyte-derived macrophages, leading to the upregulation of adhesion molecules, thus initiating a pro-inflammatory cascade in humans. TREM2, an immune receptor implicated in several diseases, is susceptible to antagonistic actions by HSP60, as evidenced in N9 microglial cell lines. This observation underscores it as a promising target for therapeutic intervention in inflammatory diseases [139]. The release of secreted HSP60 in response to inflammation has been observed in blood, either through the classical (ER and Golgi vesicles) or non-classical (by lipid raft–exosome) pathways, in various cancers and cardiomyopathy [140]. For instance, in rheumatoid arthritis (RA), lupus, bowel disease, and atherosclerosis, HSP60 expression is involved in disease progression [109]. Studies have demonstrated that in rheumatoid arthritis, lupus, bowel disease, and atherosclerosis, HSP60 levels influence monocyte differentiation into macrophages, which subsequently secrete various pro-inflammatory cytokines [141]. The induced expression of HSP60 stimulates the pro-inflammatory cytokines and autoantibodies targeting self-HSP60 in certain diseases. In Hashimoto’s thyroiditis (HT), levels of HSP60 are induced in both the bloodstream and thyrocytes, including oncocytes (Hurthle cells). Moreover, human HSP60 exhibits similarity with thyroglobulin (TG) and thyroid peroxidase (TPO) sequences. Consequently, autoantibodies against TG and TPO are likely to recognize HSP60, potentially contributing to the pathogenesis of HT [142]. Activation of p38 MAPK in hepatocytes induces the release of HSP60 and ROS from mitochondria, subsequently activating cytosolic IκB and NF-κB. This cascade leads to inflammation and oxidative stress. Avicularin, a flavonoid found in citrus fruits, suppressed the expression of HSP60, NLRP3, pIκB, and p-p65, leading to the inhibition of inflammasome formation and consequently reducing IL-1β and mitigating Pb-induced liver injury in ICR mice [110]. Consequently, T cells recognize HSP60 self-peptides in the blood samples, signifying its pivotal role in inflammatory and autoimmune conditions. In an effort to identify and assess an epitope of HSP60 for mitigating pro-inflammatory cytokine production and autoreactive T-cell responses, an N-terminal sequence (amino acids 90–109) was identified, exhibiting 100% conservation across various species. This sequence was modified into an altered peptide ligand (APL) by introducing a single mutation replacing Asp-18 with Leu. The genetic alteration increased its affinity with MHC-II, and as a result, it was approved for emergency clinical use during the COVID-19 pandemic under the name Jusvinza by the Cuban regulatory authority. Jusvinza has been shown to increase the population of FOXP3+ regulatory T cells (Treg) while reducing the level of pro-inflammatory cytokines. Specifically, it decreases the production of several cytokines, including IL-17, IL-6, IL-10, and TNF-α, as well as IFN-γ in rheumatoid arthritis (RA) patients and induces Tregs in certain experimental models (Table 2) [111]. Consequently, HSP60-derived APLs hold promise as a treatment option for various autoimmune diseases such as RA, diabetes, and atherosclerosis, as well as infections including SARS-CoV-2 and other inflammatory conditions [109]. Thus, HSP60-derived peptides may be used in regulating inflammatory and pro-inflammatory cytokines.

Notably, HSP60 triggers the activation of macrophages by binding to the TLR4 receptor, thereby initiating the synthesis of inflammatory cytokines through the activation of NF-κB and NLRP3 signaling pathways [112]. This hypothesis was supported by a study involving di-2-ethylhexyl phthalate (DEHP), a plasticizer known to affect spleen function and human health through various mechanisms. In vivo experiments with mice exposed to DEHP resulted in stress responses, leading to elevated expression of HSP60. These increased levels of HSP60 trigger an inflammatory response by binding to the TLR4 receptor, inducing NF-κB/NLRP3-dependent inflammatory signaling, ultimately resulting in pyroptosis in the mouse spleen. This suggests that HSP60 plays a critical role in inflammatory responses and could serve as a promising target in the treatment of inflammatory diseases [113]. Additionally, HSP60 maintains regulatory T cells positive for CD4, CD25, and Foxp3 [143]. In diabetes-induced neuroinflammation, HSP60 activates microglia and astrocytes, leading to the production of pro-inflammatory cytokines such as IL-1β, IL-6, and TNF-α through signaling pathways like ERK-1/2, JNK, and NF-κB. Hydroxytyrosol (HT), a polyphenol abundantly found in olive oil, having anti-inflammatory, antimicrobial, and anticancer properties, reduces HSP60 expression, resulting in a significant reduction in inflammation in cancer [144,145,146]. Additionally, HSP60 has been shown to prevent inflammation-induced cell death in rheumatoid arthritis (RA) by promoting the secretion of anti-inflammatory cytokines IL-4 and IL-10 at the site of inflammation in the bone [114,115]. 

### 4.5. Role of HSP60 in Various Cancers

HSP60 exhibits differential expression in various cancers and plays a dual role. HSP60 is overexpressed in most cancers, such as colorectal cancer, non-small-cell lung cancer (NSCLC), breast cancer, hepatocellular carcinoma (HCC), oral cancer [147], and esophageal squamous-cell carcinoma (ESCC), whereas in bladder cancer and clear-cell renal carcinoma, HSP60 levels are significantly reduced [148]. Notably, there is no significant difference in the expression of HSP60 reported in skin cutaneous melanoma (SKCM), thyroid carcinoma (THCA), and thymoma (THYM). In neuroblastoma, a pediatric tumor, HSP60 is upregulated compared to normal cells; it provides cellular protection by stabilizing survivin via binding with cell cycle and apoptosis regulator 2 (CCAR2) and contributes to the survival of neuroblastoma cells (Table 2) [116]. Conversely, loss of HSP60 expression in normal cells increases p53 expression and activates p53-dependent apoptosis, and finally, cell death. This underscores the complex and context-dependent role of HSP60 in cancer development and progression [149]. The expression of HSP60 in many cancers is dynamic, with induced expression reported in various tumors, including adrenal tumors, breast cancer, bronchial, exocervical, ovarian, and prostate cancers [80]. Downregulation of HSP60 has been linked to the development and progression of bronchial carcinogenesis [150]. Additionally, an increased expression is observed in lung adenocarcinoma with grade III compared to grades I and II. This indicates that increased expression of HSP60 may be a promising marker for lung cancer [81,151]. In tumor cells, HSP60 is released via exosomes and can interact with peritumoral cells and enter into the bloodstream. Likewise, in thyroid papillary carcinoma (PC), HSP60 levels were induced in the cytoplasm and the plasma cell membrane. Following their accumulation within the cell, HSPs can be actively released into the extracellular space and into the bloodstream. The extracellular HSP60 levels in exosomes were significantly higher compared to those in exosomes from post-surgery papillary carcinoma patient’s and patients with benign goiter (BG), suggesting their potential as biomarkers for clinical applications [152]. In colon cancer, HSP60 is localized in the pericellular interstitium of affected tissue on macrophages and NK cells and is released into circulation through HSP-EVs. Removal of tumor tissue reduces circulating HSP60 levels, suggesting its potential as a diagnostic marker for colon cancer and other cancers [153,154]. 

Guo et al. demonstrated that knocking down HSP60 in a colorectal cancer cell line resulted in inhibited cell proliferation both in vitro and in nude mouse xenografts. Additionally, depletion of HSP60 disrupted mitochondrial protein homeostasis and led to adenine accumulation. This accumulation activated the AMPK signaling pathway, subsequently inhibiting protein translation and cell proliferation [117]. Another study also indicated through bioinformatic analyses of data from the Cancer Genome Atlas (TCGA) that elevated expression of HSP60 in colorectal cancer (CRC) is associated with poor prognosis. HSP60 appears to have a dual role in CRC, exerting both tumor-suppressive and tumor-promoting effects. Analysis of TCGA data suggests an association between HSP60 expression levels and p53 expression, which may influence survival rates in CRC patients categorized into high and low HSP60 expression groups. HSP60 expression is correlated with an aggressive phenotype in prostate cancer (PCa). A recent study unveiled that inhibiting HSP60–ClpP interactions using small-molecule inhibitors such as DCEM1 suppresses Myc signaling and ATP production, causing mitochondrial dysfunctions. This inhibition leads to the suppression of PCa growth and prevents PCa recurrence [118]. Further investigation is warranted to elucidate this relationship [119]. 

**Figure 3:**
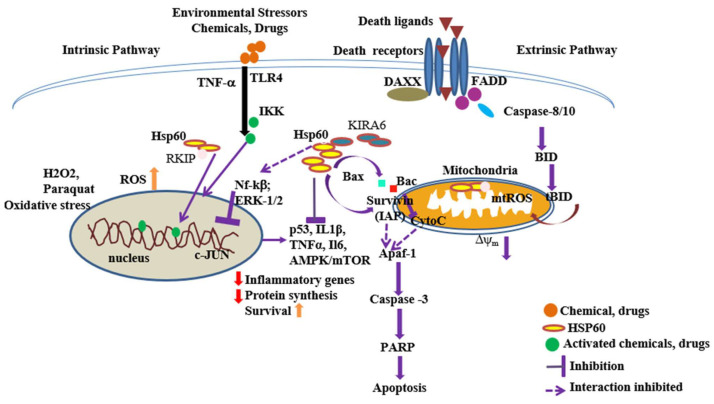
The diagram depicts the intricate interplay between HSP60 and various cellular proteins, such as p53, Nf-kβ, IKK, Bax, and survivin, influencing critical cellular processes like tumorigenesis, metastasis, inflammation, and apoptosis regulation. It illustrates how diverse stressors and stimuli, both chemical and physical, trigger downstream signaling pathways that govern protein-folding and cell-death mechanisms. Ultimately, mitochondrial dysfunction and compromised membrane integrity lead to the initiation of apoptosis-mediated cell death. The colored symbols represent interactors: orange circles, chemical stressors and drugs; green circles, activated molecules bind to DNA; marron triangles, death ligands; violet circles, fas-associated protein death domain; sky blue ovals, caspase-8; green box, BAX; red box, BAK [5].

The expression of HSP60 depends on the tissue type and the tumor microenvironment. HSP60 binds to various molecules in the cytosol, such as Bax and Bak, inhibiting the release of cytochrome C from mitochondria and preventing apoptosis in several tumors [120]. Recently, it has been demonstrated that HSP60 binds to survivin, the smallest member of the inhibitor of the apoptosis protein (IAP) family. This interaction takes place in both the cytoplasm and mitochondria, promoting cell survival [155]. Notably, in oral squamous-cell carcinoma, HSP60 levels are significantly elevated compared to adjacent control tissues. Depletion of mitochondrial HSP60 in tumor cells disrupts the stability of survivin, an anti-apoptotic protein, culminating in induced apoptosis (Table 2) [156,157]. Conversely, overexpression of HSP60 stabilizes the mitochondrial survivin complex, thereby promoting cancer cell survival (Figure 3) [155,158]. The specific molecular mechanism of the HSP60–survivin complex requires further investigation to elucidate its role in cancers. In ameloblastoma, a rare odontogenic neoplasm of mandible and maxilla. Immunohistochemistry revealed elevated levels of HSP60 within the plasma membrane of the neoplastic cells. Intriguingly, robust expression of HSP60 was observed within the nuclei of epithelial cells in peripheral ameloblastoma, implying a potential role in gene expression regulation [159]. 

HSP60 levels are context-dependent and vary among different cancer types and tissues compared to normal tissue. In hepatocellular carcinoma (HCC), the interaction between survivin and HSP60 in the cytosol, demonstrating a positive correlation, facilitates cell survival. Mifepristone, known for its antagonistic effects on both the progesterone receptor (PR) and the glucocorticoid receptor (GR), has exhibited antiproliferative properties across various cancers. Specifically, mifepristone induces cell cycle arrest at the G1 phase and early-stage apoptosis. Interestingly, mifepristone does not diminish the total amount of HSP60 but rather induces its translocation from mitochondria to the cytosol by disrupting the mitochondrial membrane potential (ΔΨm). Consequently, this relocation enhances the formation of a complex between glucocorticoid receptor (GR)–HSP60–survivin, leading to the degradation of survivin via the ubiquitous proteasomal pathway (UPP) [160]. These findings underscore the potential therapeutic significance of inhibiting HSP60 to suppress the growth of HCC. A report on HCC revealed that the interaction between Raf kinase inhibitor protein (RKIP), a negative regulator of MAPK, and HSP60 is influenced by TPA-induced oxidation of HSP60 in mitochondria. Additionally, PKCδ activation leads to induced mitochondrial ROS (mtROS), causing oxidation of HSP60 in the cytosol, which relieves RKIP, promoting the translocation of HSP60/MAPK from mitochondria to the cytosol. Consequently, MAPK is activated in both mitochondria and the cytosol. This process ultimately regulates the gene expression, leading to migration and G1 cell cycle arrest in HCC [121]. Targeting HSP60 may hold promise for reducing adverse effects and drug resistance in many tumor and cancer cells [81,161]. 

HSP60 expression is higher in nasopharyngeal carcinoma (NPC) and contributes to enhanced cell survival. PQR309, an inhibitor of PI3K signaling, combined with gemcitabine, demonstrates a synergistic effect, effectively inhibiting NPC tumor activity by reducing STAT3-mediated HSP60 expression. The STAT3-binding site on the HSP60 promoter regulates its expression, reinforcing the efficacy of PQR309 in suppressing NPC progression in patients [122]. Similarly, in ovarian cancer (OC), the role of HSP60 remains ambiguous. A study conducted on ovarian cancer cell lines revealed that suppressing HSP60 significantly enhances cell proliferation and migration in vitro. However, these are inconsistencies in outcomes across different cell lines. Knockdown of HSP60 in OC cell lines (such as SKOV3 and OVCAR3) results in downregulation of various mitochondrial proteins, including 3-oxoacyl-ACP synthase (OXSM), which plays a role in lipoic acid (LA) biosynthesis and metabolism [123]. In another study on OC, hormone-sensitive lipase (HSL) and lipid-mobilizing factor (LMF) were detected in the serum and ascites of the patients, in contrast to their levels in a normal control group. Nonetheless, further in vivo analysis is necessary to investigate its potential as a diagnostic and therapeutic target in ovarian cancers. Proteomic analyses of mitochondria in ovarian cancer samples showed an increase in both HSP60 and phosphorylated HSP60 at Ser70, which influence the acyl-coA dehydrogenase (ACADS) enzyme activity, resulting in alterations in fatty acid oxidation and energy supply [38]. The ATP-dependent “foldase” activity of HSP60 may induce conversion of pro-caspase-3, stimulating apoptosis through the Fas-independent pathway in Jurkat cells. In cancer cells, inhibitors of HSP60 such as Mizoribine and KIRA6 inhibit the activation of CXCL8, a pro-inflammatory chemokine, suggesting a role for HSP60 as an anti-inflammatory regulator in response to immunogens. KIRA6 is a prototype of ATP-binding compounds disrupting the oligomerization and RNase activities of IRE1α and suppresses the HSP60–IKKβ-mediated activation of Nf-kB-mediated pro-inflammatory response in cancers (Table 2) [124]. Therefore, this indicates that HSP60 acts as an anti-inflammatory regulator evoked by immunogens (Figure 3). These findings indicate that HSP60 has potential applications both as an early diagnostic biomarker and a therapeutic target in cancers and immune disorders. 

### 4.6. Role of HSP60 in Neurodegeneration

In neurodegenerative diseases, various stresses cause protein aggregation and the accumulation of unfolded/misfolded proteins, resulting in synaptic loss and neuronal death. Oxidative stress and elevated ROS levels contribute to mitochondrial dysfunction in Alzheimer’s disease (AD) and Parkinson’s disease (PD) pathologies [162]. Numerous studies indicate that HSPs play a role in protein metabolism and the aggregation of both Aβ and tau [163]. Studies have shown that the release of secretory HSP60 in extramitochondrial sites such as the interstitial space, cell membrane, and biological fluids contributes to neuroinflammation [164]. The presence of HSP60 in the extracellular space induces neuroinflammation and results in neuronal death. Thereby, the inhibition of HSP60 expression and its release in the extracellular area may have the potential to ameliorate the inflammation and decrease neuronal cell death [125]. In AD, protein aggregation plays a crucial role in disease progression. Clinical samples from both sporadic and familial AD cases have shown the colocalization of HSP60 and Aβ in the frontal cortex, indicating the involvement of HSP60 in the regulation of AD. In vivo and ex vivo experiments have shown that HSP60 protects neuronal cells by blocking Aβ1-42 toxicity and restoring long-term potentiation (LTP) and synaptic plasticity in mouse hippocampus [126,127]. 

Mitochondrial HSP60 facilitates the removal of Aβ from the mitochondrial matrix by triggering the unfolded protein response (UPR), which, in turn, upregulates several key genes involved in mitochondrial proteostasis. Additionally, mitochondrial HSP60 has been found to associate with other heat shock proteins, such as HSP70 and HSP90, as well as extracellular and intracellular amyloid-β proteins. The accumulation of amyloid-β inhibits the electron transport cycle (ETC), particularly affecting complex IV, leading to mitochondrial dysfunction. This dysfunction results in the release of cytochrome C, ultimately causing cell death. In the context of neurodegenerative diseases like Alzheimer’s, in vitro studies using human neuroblastoma cells (SH-SY5Y) have shown that overexpression of HSP60, alone or in combination with HSP70 and HSP90, can mitigate cytotoxicity induced by intracellular β-amyloid. This suggests that mitochondrial HSP60 may offer protection against damage to complex IV and pyruvate dehydrogenase (PDH) induced by intracellular Aβ, thereby maintaining ATP levels in mitochondria [92]. Consequently, mitochondrial HSP60 could serve as a potential therapeutic target for early-stage neurodegenerative diseases, where intracellular Aβ accumulation is a significant pathological factor [165]. HSP60 also regulates Aβ accumulation and aggregate formation in neurons [166]. 

Investigations have shown that human HSP60 (HSPD1) binds to Aβ oligomers, reducing neurotoxicity by inhibiting their interaction with the membrane [106,167]. In vitro, mitochondrial HSP60 binds to Aβ oligomers, mitigating Aβ-mediated mitochondrial dysfunction and neuronal death [126]. In mitochondrial misfolded/ unfolded protein activate the unfolded protein response (UPR), which induces several key genes such as ClpP, Lonp1, txn2 along with HSPD1, and HSPE1 in the frontal cortex in sporadic or familial AD involved in mitochondrial proteostasis [128]. These studies suggest that mitochondrial HSP60 has the beneficial effect of managing mitochondrial UPR and modulating the formation of Aβ, resulting in the rescue of mitochondrial activity in neuroblastoma cells and primary cortical neurons [165]. Many reports suggest that HSP60 levels are critical for regulating mitochondrial proteins and apoptosis in various neuronal diseases. However, further investigation is necessary to better understand the role of HSP60 in human diseases.

### 4.7. Chemical Inhibitors and Modulators of HSP60

HSP60 plays a crucial role in various cellular processes, including protein folding and refolding. Dysregulation of HSP60 has been implicated in numerous diseases. Consequently, numerous pharmacological inhibitors or modulators targeting HSP60 are being actively evaluated as novel anticancer agents. These inhibitors fall into two main categories: (1) regulate ATPase activity and hydrolysis, and (2) bind to cysteine residue at the N-terminal of HSP60 or inhibit its interaction with HSP10. A few inhibitors, such as mizoribine [168], myrtucommulone A [169], and pyrazolopyrimidines EC3016 [170], interact with cysteine residues, leading to a significant decrease in HSP60 levels. Another promising inhibitor is copper complex 1 [171], which competitively inhibits ATP and affects protein refolding. Some inhibitors such as epolactaene, epolactaene tert-butyl ester [172], stephacidin B, and avrainvillamide [173] interact with Cys442, along with β-lactams, bind to the N-terminal regions of HSP60. Others like suvanine [174], gold (III) porphyrin complexes [175], and o-carboranylphenoxyacetanilide derivative 2 [176] act on non-ATP-competitive chaperonin inhibitors. Gossypol and 4-hydroxynonenal significantly affect HSP60’s activity by targeting thiol groups [177]. 

Natural modulators, such as aqueous extract of Terminalia arjuna [178], oxymatrine [179], β-caryophyllene [180], and nonactin [97,181] have also been found to modulate the HSP60 levels and activity. However, the precise mechanism of action for most HSP60 inhibitors remains ambiguous [182]. For therapeutic use, some inhibitors have shown promise in treating certain cancers. Small-molecule inhibitor KHS101 inhibits mitochondrial HSP60 in glioblastoma multiforme (GBM) [183]. Recently, bis-aryl-α,β unsaturated ketones [184], mifepristone [160], and KIRA6 [124] have all been found to reduce HSP60 levels in specific types of cancer. Doxorubicin reduces HSP60 levels through acetylation [185], while suberoylanilide hydroxamic acid (SAHA) lowers HSP60 levels through nitration-post-translational modification, disrupting its interaction with HSP10 and facilitating its release into the intracellular space via exosomes, thereby enhancing its interaction with the immune system [186]. Curcumin and its derivatives display inhibitory activity on HSP60-induced cell proliferation in neuroblastoma cells, though the exact mechanism remains unknown [187]. New HSP60 inhibitors have been developed, targeting the interaction sites between mitochondrial HSP60 and cochaperone HSP10 [166]. The impact of HSP60 inhibitors on molecular chaperone activity or post-translational modifications of HSP60 could potentially enhance the efficacy of cancer treatment [161]. More research is needed to determine the potential applications of HSP60 inhibitors in human diseases such as cancer and neurodegeneration. 

## 5. Conclusions and Future Prospects 

This review delves into the expression patterns of HSP60-related genes/proteins and their probable role in cellular protection against thermal stresses, as well as in normal growth and differentiation processes spanning from prokaryotes to eukaryotes. The HSP60 gene demonstrates varying expression levels, exhibiting both non-inducible and inducible patterns throughout different developmental stages. Its distinctive features, including localization within cytoplasmic and nuclear regions apart from mitochondria, render it particularly intriguing. While initial observations suggest that HSP60 may not be heat-inducible, previous studies have evidenced its induction under high-temperature stress, particularly in specific tissue or cell types, implying its involvement in stress regulation. Nonetheless, the inducible promoter region exhibits variation among species, necessitating further in vivo and in vitro investigation to elucidate its inducible function under diverse developmental and stress conditions [45]. Such studies would provide insights into its role in managing high-temperature stress and other stressors. 

Interestingly, HSP60 expression remains unaffected even after treatment with transcriptional and translational inhibitors like actinomycin D and cycloheximide, suggesting a regulatory mechanism distinct from regular transcription processes. The elevated levels of HSP60 mRNA or protein across various tissue types hint at its potential functions in mitigating high-temperature stress and preventing cellular damage induced by oxidative stress and environmental stressors [44,45,67]. Similar to other heat shock proteins, HSP60 also aids in folding and assembling target proteins under normal conditions. Moreover, the accumulation of unfolded proteins under various environmental stress conditions may induce HSP60 expression [188]. Cellular thermal sensitivity is influenced by factors like pH, elevated temperature, cold shock, and heavy metals, among others, necessitating the induced transcription of HSPs as a protective mechanism to maintain cellular homeostasis. HSP60, rather than serving its conventional functions, exhibits promise for oral vaccine development. Specifically, HSP60 from *Brucella abortus* demonstrates a strong affinity towards human prion proteins, including PrP125, PrP174, and PrP180, expressed on microfold cells (M cells) overlying Peyer’s patch (PP) [189]. This interaction with recombinant proteins assists in developing oral vaccines targeting various bacteria, viruses, and parasites, thereby stimulating mucosal immune responses. Consequently, these vaccines promote the secretion of IgA antibodies against pathogens at the infection sites. HSP60’s functions are extended to other physiological processes, including its involvement in preserving steroidogenesis within mitochondria, which is crucial for progesterone (P4) synthesis and early pregnancy maintenance [190]. Additional investigation is warranted to elucidate its precise role in P4 synthesis and the maintenance of pregnancy. 

The thermal sensitivity or stress response of different cell types may vary depending on physiological states or inducers, which, in turn, affects the expression patterns of responsive genes. The HSP60 class of heat shock proteins is implicated in numerous cellular functions, including proper protein folding, cell signaling, apoptosis, cell differentiation, and development [80]. While the role of HSP70 in thermotolerance is well-established [191,192], only a few reports suggest the likely involvement of HSP60 in thermotolerance [44,45,46]. Hence, HSP60 emerges as a crucial player in development and may hold significance in regulating various diseases such as cancer, atherosclerosis, and neurodegeneration, potentially paving the way for the development of early diagnostic markers and treatment strategies for these conditions.

## Figures and Tables

**Table 1:** HSP60 grouping, representative chaperones, localization, and functions.

Groups	Chaperonins	Organisms	Localization	Characteristics
Group I	GroEL/GroEs HSP60/HSP10, Cpn60, HSPD1/E	Prokaryotes, Eukaryotes	Cytoplasm, Mitochondria, Nucleus, and Chloroplast	Homo-oligomeric, GroES/HSP10-dependent, seven subunits per ring, assist folding
Group II	TRiC/CCT, TCP1,	Archaea, Eukaryotes cytosol	Cytosol	Hetero-oligomeric, GroES/HSP10-independent, eight to nine subunits per ring, binds to actin and stabilizes the cytoskeleton

**Table 2:** HSP60’s roles in the regulation of various human diseases.

Stress Protein	Types of Disease	Role of HSP60	References
Molecular chaperonin HSP60/cpn60	Metabolic diseases Diabetes	Inhibition of MEK/ERK induces HSP60 levels in insulin resistance	[93]
		Reducing HSP60 promotes hyperglycemia in the brain and mitochondrial dysfunctions	[94]
		Interaction with Sirt and mitochondria energy metabolism	[97,98]
	Infectious diseases Gastrointestinal diseases, Takayasu’s arteritis	Cell surface expression of HSP60 in bacterial disease	[99,100]
		Induced HSp60 in gastric dysplasia and gastric cancer	[101,102]
		Regulate viral RNA replication in foot-and-mouth disease	[103]
	Cardiovascular diseases and atherosclerosis	Activation of TLR2 and TLR4 pathways	[104,105]
		Mitochondrial dysfunctions and apoptosis in cardiomyocytes	[106]
		Induced expression of inflammatory cytokines and TNF-α secretion	[107,108]
	Inflammatory diseases	HSP60 triggers Cytokine signaling and their release	[109]
		Hepatocyte activation of p38 MAPK and IκB and NF-κB in response to oxidative stress in mitochondria	[110]
		Interaction with MHC-II, reducing pro-inflammatory cytokines	[111]
		Activates NF-κB and NLRP3 signaling pathways	[112,113]
		In RA, it promotes secretion of IL-4 and IL-10	[114,115]
	Cancers almost all types of cancer	Interact with survivin and stabilize it in neuroblastoma	[116]
		Disruption of mitochondrial protein homeostasis in colorectal cancer and prostate cancer promotes cell growth	[117,118,119]
		Interact of HSP60 with cytochrome C and DAXX; linked with its pro- and anti-apoptotic functions	[120]
		Regulate the cell cycle and mitoROS in HCC	[121]
		STAT3 dependent role in NPC	[122]
		Regulation of lipogenesis in OC	[123]
		Suppresses pro-inflammatory response in cancer via IKKβ-mediated activation of Nf-kB	[124]
	Neurodegenerative diseases	Pro-inflammatory cytokines IL-1β, IL-6, and TNF-α bind to Aβ oligomers	[125]
		Regulate the LTP and synaptic plasticity in the frontal cortex	[126,127]
		Maintaining the complex IV and PDH	[92]
		Regulate the expression of UPR genes	[128]

## Abbreviations

Cpn60	Chaperonin 60
HSP60	Heat Shock Protein 60
HSP70	Heat Shock Protein 70
HSP90	Heat Shock Protein 90
CCT	Chaperonin-Containing TCP-1
TRIC	TCP-1 Ring Complex
TCP1	Tailless Complex Polypeptide-1
ROS	Reactive Oxygen Species
mtHSP60	Mitochondrial Chaperone, 60 kDa
ER	Endoplasmic Reticulum
FBs	Fat Bodies
MTs	Malpighian Tubules
SG	Salivary Gland
IKK	IκB Kinase
ERKs	Extracellular Signal-Regulated Kinases
TLRs	Toll-Like Receptors
VSMCs	Venous Smooth Muscle Cells
ETC	Electron Transport Chain
PDH	Pyruvate Dehydrogenase
RA	Rheumatoid Arthritis
JNKs	c-Jun N-Terminal Kinases
AD	Alzheimer’s Disease
PD	Parkinson’s Disease
ESC	Embryonic Stem Cell
EB	Embryoid Body
MM	Multiple Myeloma
PCs	Plasma Cells
TCGA	The Cancer Genome Atlas
M cells	Microfold cells
NPC	Nasopharyngeal Carcinoma
